# (*E*)-4-[(4-Methoxy­phen­yl)imino­meth­yl]-*N*,*N*-dimethyl­aniline

**DOI:** 10.1107/S1600536809003869

**Published:** 2009-02-06

**Authors:** Leela Sundararaman, Hema Ramu, Ramamurthi Kandaswamy, Helen Stoeckli-Evans

**Affiliations:** a School of Physics, Bharathidasan University, Tiruchirappalli 620 024, India; b Reader in Physics, Seethalakshmi Ramaswamy College (Autonomous), Tiruchirappalli 620 002, India; c Institute of Physics, University of Neuchatel, rue Emile-Argand 11, CH-2009 Neuchatel, Switzerland

## Abstract

The title compound, C_16_H_18_N_2_O, an *N*-benzyl­ideneaniline derivative with substituents on both aromatic rings, crystallizes with two independent mol­ecules (*A* and *B*) in the asymmetric unit. Both mol­ecules exist in the *E* configuration. The dihedral angle between the two aromatic rings is 8.20 (5)° for mol­ecule *A* and 12.52 (6)° for mol­ecule *B*, and the imino C—N torsion angle (τ) is 7.1 (2)° for mol­ecule *A* and −14.7 (2)° for mol­ecule *B*. In the crystal structure, mol­ecules are arranged in stacks which propagate along the *a-*axis direction. The crystal structure is stabilized by a number of weak C—H⋯π inter­actions.

## Related literature

For the conformational analysis of similar compounds, see: Bernstein *et al.* (1981 ▶); For the structures of related compounds, see: Clegg *et al.* (1996 ▶), Ahmet *et al.* (1994 ▶).
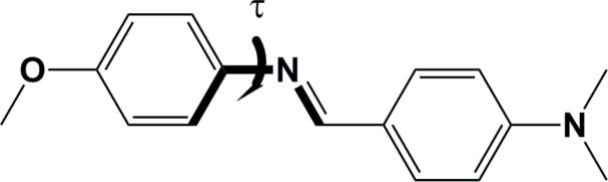


## Experimental

### 

#### Crystal data

C_16_H_18_N_2_O
*M* *_r_* = 254.32
Monoclinic, 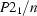

*a* = 9.6638 (6) Å
*b* = 28.5819 (15) Å
*c* = 9.9729 (7) Å
β = 98.741 (5)°
*V* = 2722.6 (3) Å^3^
*Z* = 8
Mo *K*α radiation
μ = 0.08 mm^−1^
*T* = 173 (2) K
0.33 × 0.30 × 0.28 mm

#### Data collection

Stoe IPDS-2 diffractometer
Absorption correction: none
23112 measured reflections
5128 independent reflections
3960 reflections with *I* > 2σ(*I*)
*R*_int_ = 0.036

#### Refinement

*R*[*F* ^2^ > 2σ(*F* ^2^)] = 0.035
*wR*(*F* ^2^) = 0.093
*S* = 1.04
5128 reflections
350 parameters
H-atom parameters constrained
Δρ_max_ = 0.16 e Å^−3^
Δρ_min_ = −0.15 e Å^−3^

### 

Data collection: *X-AREA* (Stoe & Cie, 2006 ▶); cell refinement: *X-AREA*; data reduction: *X-RED32* (Stoe & Cie, 2006 ▶); program(s) used to solve structure: *SHELXS97* (Sheldrick, 2008 ▶); program(s) used to refine structure: *SHELXL97* (Sheldrick, 2008 ▶); molecular graphics: *PLATON* (Spek, 2003 ▶); software used to prepare material for publication: *SHELXL97*.

## Supplementary Material

Crystal structure: contains datablocks I, global. DOI: 10.1107/S1600536809003869/nc2130sup1.cif

Structure factors: contains datablocks I. DOI: 10.1107/S1600536809003869/nc2130Isup2.hkl

Additional supplementary materials:  crystallographic information; 3D view; checkCIF report

## Figures and Tables

**Table 1:** Intermolecular C—H⋯π interactions (Å, °)

D—H⋯*Cg*	D—H	H⋯*Cg*	D—H⋯*Cg*
C3—H3⋯*Cg*3	0.95	2.63	152
C6—H6⋯*Cg*3^i^	0.95	2.81	142
C10—H10⋯*Cg*4	0.95	2.88	145
C13—H13⋯*Cg*4^i^	0.95	2.73	137
C30—H30⋯*Cg*2^ii^	0.95	2.94	136
C34—H34*A*⋯*Cg*1^iii^	0.95	2.96	114
C36—H36*A*⋯*Cg*1^iv^	0.95	2.91	156

Centroids: *Cg*1 = ring C1–C6; *Cg*2 = ring C8–C13; *Cg*3 = ring C21–C26; *Cg*4 = ring C28–C33. Symmetry codes: (i) 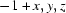; (ii) 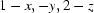; (iii) 
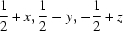
; (iv) 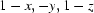.

**Fig. 1.**
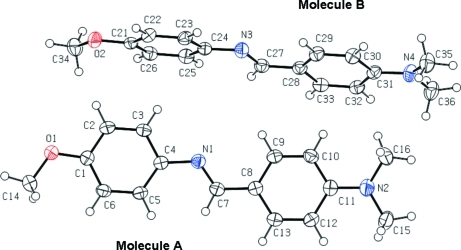
View of the two independent molecules (A & B) of the title compound, showing the atom-labeling scheme and displacement ellipsoids drawn at the 50% probability level (H atoms are represented by circles of arbitrary radii).

**Fig. 2.**
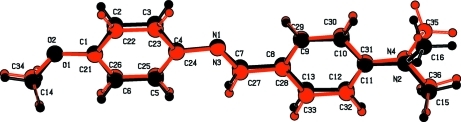
The Auto-Fit (Spek, 2003) diagram of molecules A (in black) and B (in red).

**Fig. 3.**
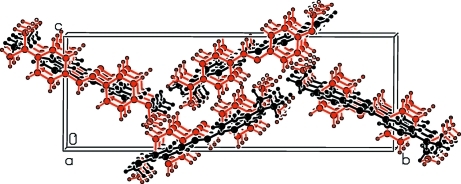
A view along the a axis of the crystal packing of the title compound [molecule A is in black, molecule B is in red].

## supplementary crystallographic information

### Comment

Interest in the optical properties of *N*-benzylideneaniline derivatives is focused on potential applications, such as liquid crystalline and non-linear optical properties. The second harmonic generation properties have been widely studied (Clegg *et al.*, 1996 and references therein). Bernstein *et al.* (1981) have shown by *ab initio* calculations on benzylideneaniline and related molecules, that rotations about the Ph—N bond of up to 45° from a planar conformation are stabilizing, while rotations about the Ph—C bond are destabilizing, and the most stable free-molecule conformation is non-planar. In contrast, benzylideneaniline derivatives with substituents on one or both aromatic rings display a range of conformations in the solid state (Clegg *et al.*, 1996).

The asymmetric unit of the title compound contains two crystallographically independent molecules (A and B), as shown in Fig. 1. Both molecules exist in the E-configuration and their bond lengths and angles are comparable to those in related structures (Ahmet *et al.*, 1994; Clegg *et al.*, 1996). The dihedral angle between the two aromatic rings is 8.20 (5)° for molecule A and 12.52 (6)° for molecule B, and the imino C—N torsion angle (τ) is 7.05 (18)° in molecule A and -14.68 (18)° in molecule B (see Table 1). This slight difference in the conformation of the two molecules is illustrated by the Auto-Fit diagram (r.m.s. Bond Fit = 0.0028 Å; r.m.s Angle Fit = 0.566°; Spek, 2008), as shown in Fig. 2.

In the crystal structure molecules A and B (*i.e.* the mean planes through all the non-H atoms in each molecule) are inclinded to one another by 68.8 (5)°. The molecules pack in stacks along the a direction (Fig. 3), and the crystal structure is stabilized by a number of C—H···π interactions (Table 2).

### Experimental

The title compound (CAS Registry No. 97221–11-9) was prepared by the reaction of 4-dimethylaminobenzaldehyde (2 mmol) with 4-methoxyaniline (2 mmol) in absolute ethanol (10 ml) for one day at room temperature. The solvent was evaporated and the product was recrystallized from acteone, giving yellow block-like crystals suitable for X-ray analysis.

### Refinement

H atoms were included in calculated positions and treated as riding atoms: C—H = 0.95 - 0.98 Å, with *U*_iso_ = 1.2 or 1.5*U*_eq_(parent C-atom).

### Crystal data

**Table:**

C_16_H_18_N_2_O	*F*(000) = 1088
*M**_r_* = 254.32	*D*_x_ = 1.241 Mg m^−^^3^
Monoclinic, *P*2_1_/*n*	Mo *K*α radiation, λ = 0.71073 Å
Hall symbol: -P 2yn	Cell parameters from 18324 reflections
*a* = 9.6638 (6) Å	θ = 1.4–26.1°
*b* = 28.5819 (15) Å	µ = 0.08 mm^−^^1^
*c* = 9.9729 (7) Å	*T* = 173 K
β = 98.741 (5)°	Block, yellow
*V* = 2722.6 (3) Å^3^	0.33 × 0.30 × 0.28 mm
*Z* = 8	

### Data collection

**Table:**

Stoe IPDS-2 diffractometer	3960 reflections with *I* > 2σ(*I*)
Radiation source: fine-focus sealed tube	*R*_int_ = 0.036
graphite	θ_max_ = 25.7°, θ_min_ = 1.4°
Detector resolution: 6.67 pixels mm^-1^	*h* = −11→10
φ and ω scans	*k* = −34→34
23112 measured reflections	*l* = −12→12
5128 independent reflections	

### Refinement

**Table:**

Refinement on *F*^2^	Secondary atom site location: difference Fourier map
Least-squares matrix: full	Hydrogen site location: inferred from neighbouring sites
*R*[*F*^2^ > 2σ(*F*^2^)] = 0.035	H-atom parameters constrained
*wR*(*F*^2^) = 0.093	*w* = 1/[σ^2^(*F*_o_^2^) + (0.057*P*)^2^ + 0.0294*P*] where *P* = (*F*_o_^2^ + 2*F*_c_^2^)/3
*S* = 1.03	(Δ/σ)_max_ < 0.001
5128 reflections	Δρ_max_ = 0.16 e Å^−^^3^
350 parameters	Δρ_min_ = −0.15 e Å^−^^3^
0 restraints	Extinction correction: *SHELXL97* (Sheldrick, 2008), Fc^*^=kFc[1+0.001xFc^2^λ^3^/sin(2θ)]^-1/4^
Primary atom site location: structure-invariant direct methods	Extinction coefficient: 0.0072 (11)

### Special details

**Table:**

Geometry. Bond distances, angles *etc*. have been calculated using the rounded fractional coordinates. All su's are estimated from the variances of the (full) variance-covariance matrix. The cell e.s.d.'s are taken into account in the estimation of distances, angles and torsion angles
Refinement. Refinement of *F*^2^ against ALL reflections. The weighted *R*-factor *wR* and goodness of fit *S* are based on *F*^2^, conventional *R*-factors *R* are based on *F*, with *F* set to zero for negative *F*^2^. The threshold expression of *F*^2^ > σ(*F*^2^) is used only for calculating *R*-factors(gt) *etc*. and is not relevant to the choice of reflections for refinement. *R*-factors based on *F*^2^ are statistically about twice as large as those based on *F*, and *R*- factors based on ALL data will be even larger.

### Fractional atomic coordinates and isotropic or equivalent isotropic displacement parameters (Å^2^)

**Table:**

	*x*	*y*	*z*	*U*_iso_*/*U*_eq_	
O1	−0.01231 (8)	0.26535 (3)	0.41055 (9)	0.0343 (3)	
N1	0.18794 (10)	0.09281 (3)	0.61633 (10)	0.0310 (3)	
N2	0.34868 (12)	−0.10828 (4)	0.89245 (11)	0.0409 (3)	
C1	0.02582 (12)	0.22093 (4)	0.45339 (11)	0.0277 (3)	
C2	0.16891 (12)	0.21336 (4)	0.48916 (11)	0.0302 (3)	
C3	0.21674 (12)	0.17052 (4)	0.54052 (11)	0.0289 (3)	
C4	0.12485 (12)	0.13401 (4)	0.55728 (11)	0.0273 (3)	
C5	−0.01789 (12)	0.14196 (4)	0.51769 (12)	0.0326 (4)	
C6	−0.06724 (12)	0.18490 (4)	0.46633 (12)	0.0315 (3)	
C7	0.11725 (12)	0.05582 (4)	0.62753 (11)	0.0298 (4)	
C8	0.17909 (12)	0.01367 (4)	0.69266 (11)	0.0283 (3)	
C9	0.32174 (12)	0.01043 (4)	0.74581 (12)	0.0317 (4)	
C10	0.37765 (12)	−0.02905 (4)	0.81114 (12)	0.0333 (4)	
C11	0.29289 (13)	−0.06815 (4)	0.82823 (11)	0.0307 (3)	
C12	0.15020 (13)	−0.06498 (4)	0.77430 (12)	0.0342 (3)	
C13	0.09608 (12)	−0.02504 (4)	0.70861 (12)	0.0327 (4)	
C14	−0.15718 (13)	0.27386 (5)	0.36613 (15)	0.0415 (4)	
C15	0.25698 (16)	−0.14624 (5)	0.91713 (15)	0.0501 (5)	
C16	0.48167 (14)	−0.10627 (6)	0.98195 (16)	0.0541 (5)	
O2	0.48614 (9)	0.26329 (3)	0.41654 (8)	0.0363 (3)	
N3	0.66974 (10)	0.09634 (4)	0.67327 (10)	0.0330 (3)	
N4	0.84937 (12)	−0.10741 (3)	0.91646 (10)	0.0355 (3)	
C21	0.53069 (11)	0.22039 (4)	0.46791 (11)	0.0288 (3)	
C22	0.56508 (12)	0.21854 (4)	0.60834 (11)	0.0299 (3)	
C23	0.61013 (12)	0.17706 (4)	0.67068 (11)	0.0300 (3)	
C24	0.62180 (11)	0.13625 (4)	0.59646 (11)	0.0284 (3)	
C25	0.58756 (12)	0.13886 (4)	0.45565 (12)	0.0324 (4)	
C26	0.54305 (12)	0.18050 (4)	0.39161 (11)	0.0314 (4)	
C27	0.65947 (12)	0.05552 (4)	0.62163 (12)	0.0310 (4)	
C28	0.70670 (12)	0.01390 (4)	0.69932 (12)	0.0293 (3)	
C29	0.76315 (12)	0.01597 (4)	0.83718 (12)	0.0317 (3)	
C30	0.80752 (13)	−0.02356 (4)	0.90926 (12)	0.0321 (3)	
C31	0.79938 (12)	−0.06800 (4)	0.84664 (11)	0.0288 (3)	
C32	0.73941 (12)	−0.07010 (4)	0.70913 (11)	0.0300 (3)	
C33	0.69511 (12)	−0.03003 (4)	0.63833 (12)	0.0308 (4)	
C34	0.45066 (15)	0.26764 (5)	0.27361 (13)	0.0444 (5)	
C35	0.89078 (16)	−0.10601 (5)	1.06188 (13)	0.0449 (4)	
C36	0.83689 (16)	−0.15253 (4)	0.85068 (14)	0.0433 (4)	
H2	0.23350	0.23760	0.47820	0.0360*	
H3	0.31460	0.16570	0.56510	0.0350*	
H5	−0.08250	0.11750	0.52610	0.0390*	
H6	−0.16490	0.18960	0.44000	0.0380*	
H7	0.02030	0.05580	0.59190	0.0360*	
H9	0.38130	0.03630	0.73630	0.0380*	
H10	0.47490	−0.03010	0.84540	0.0400*	
H12	0.09010	−0.09070	0.78320	0.0410*	
H13	−0.00080	−0.02400	0.67320	0.0390*	
H14A	−0.19210	0.25200	0.29320	0.0620*	
H14B	−0.20920	0.26950	0.44220	0.0620*	
H14C	−0.16990	0.30600	0.33240	0.0620*	
H15A	0.19700	−0.13620	0.98260	0.0750*	
H15B	0.19860	−0.15510	0.83180	0.0750*	
H15C	0.31320	−0.17320	0.95370	0.0750*	
H16A	0.47180	−0.08700	1.06130	0.0810*	
H16B	0.55260	−0.09240	0.93350	0.0810*	
H16C	0.51050	−0.13800	1.01150	0.0810*	
H22	0.55750	0.24580	0.66120	0.0360*	
H23	0.63380	0.17620	0.76670	0.0360*	
H25	0.59480	0.11160	0.40260	0.0390*	
H26	0.52110	0.18170	0.29550	0.0380*	
H27	0.61950	0.05210	0.52910	0.0370*	
H29	0.77090	0.04540	0.88180	0.0380*	
H30	0.84430	−0.02100	1.00290	0.0380*	
H32	0.72930	−0.09950	0.66440	0.0360*	
H33	0.65560	−0.03250	0.54540	0.0370*	
H34A	0.53210	0.25960	0.23040	0.0670*	
H34B	0.37320	0.24640	0.24110	0.0670*	
H34C	0.42220	0.29990	0.25060	0.0670*	
H35A	0.96730	−0.08350	1.08450	0.0670*	
H35B	0.81080	−0.09640	1.10520	0.0670*	
H35C	0.92240	−0.13710	1.09460	0.0670*	
H36A	0.88130	−0.15140	0.76870	0.0650*	
H36B	0.73770	−0.16050	0.82600	0.0650*	
H36C	0.88320	−0.17630	0.91270	0.0650*	

### Atomic displacement parameters (Å^2^)

**Table:**

	*U*^11^	*U*^22^	*U*^33^	*U*^12^	*U*^13^	*U*^23^
O1	0.0305 (4)	0.0322 (5)	0.0398 (5)	−0.0008 (4)	0.0042 (4)	0.0072 (4)
N1	0.0302 (5)	0.0301 (5)	0.0332 (5)	0.0002 (4)	0.0069 (4)	0.0009 (4)
N2	0.0426 (6)	0.0360 (6)	0.0439 (6)	0.0030 (5)	0.0063 (5)	0.0081 (5)
C1	0.0319 (6)	0.0292 (6)	0.0224 (5)	−0.0001 (5)	0.0059 (4)	−0.0005 (4)
C2	0.0278 (6)	0.0318 (6)	0.0320 (6)	−0.0057 (5)	0.0080 (5)	−0.0021 (5)
C3	0.0241 (5)	0.0330 (6)	0.0299 (6)	−0.0016 (5)	0.0053 (4)	−0.0030 (5)
C4	0.0282 (6)	0.0290 (6)	0.0252 (5)	−0.0020 (5)	0.0059 (4)	−0.0027 (4)
C5	0.0277 (6)	0.0306 (6)	0.0397 (7)	−0.0051 (5)	0.0060 (5)	0.0004 (5)
C6	0.0238 (5)	0.0356 (7)	0.0347 (6)	−0.0023 (5)	0.0031 (5)	−0.0002 (5)
C7	0.0286 (6)	0.0340 (7)	0.0265 (6)	−0.0007 (5)	0.0037 (5)	−0.0024 (5)
C8	0.0306 (6)	0.0306 (6)	0.0243 (5)	−0.0012 (5)	0.0066 (4)	−0.0027 (5)
C9	0.0298 (6)	0.0339 (7)	0.0326 (6)	−0.0040 (5)	0.0084 (5)	−0.0003 (5)
C10	0.0265 (6)	0.0380 (7)	0.0357 (6)	0.0010 (5)	0.0056 (5)	0.0008 (5)
C11	0.0356 (6)	0.0311 (6)	0.0267 (6)	0.0035 (5)	0.0087 (5)	−0.0017 (5)
C12	0.0355 (6)	0.0308 (6)	0.0363 (6)	−0.0058 (5)	0.0059 (5)	−0.0004 (5)
C13	0.0281 (6)	0.0357 (7)	0.0335 (6)	−0.0028 (5)	0.0024 (5)	−0.0021 (5)
C14	0.0347 (7)	0.0388 (7)	0.0492 (8)	0.0041 (6)	0.0005 (6)	0.0079 (6)
C15	0.0593 (9)	0.0327 (7)	0.0560 (9)	−0.0012 (7)	0.0018 (7)	0.0090 (6)
C16	0.0371 (7)	0.0544 (9)	0.0694 (10)	0.0074 (7)	0.0036 (7)	0.0241 (8)
O2	0.0405 (5)	0.0331 (5)	0.0344 (5)	−0.0009 (4)	0.0030 (4)	0.0086 (4)
N3	0.0348 (6)	0.0309 (6)	0.0339 (5)	0.0029 (4)	0.0076 (4)	0.0024 (4)
N4	0.0478 (6)	0.0270 (5)	0.0318 (5)	0.0025 (5)	0.0069 (5)	−0.0020 (4)
C21	0.0220 (5)	0.0314 (6)	0.0331 (6)	−0.0040 (5)	0.0048 (5)	0.0059 (5)
C22	0.0331 (6)	0.0266 (6)	0.0305 (6)	−0.0045 (5)	0.0069 (5)	−0.0006 (5)
C23	0.0312 (6)	0.0324 (6)	0.0263 (6)	−0.0046 (5)	0.0042 (5)	0.0015 (5)
C24	0.0245 (6)	0.0299 (6)	0.0316 (6)	−0.0012 (5)	0.0067 (4)	0.0014 (5)
C25	0.0316 (6)	0.0352 (7)	0.0311 (6)	0.0000 (5)	0.0071 (5)	−0.0037 (5)
C26	0.0283 (6)	0.0397 (7)	0.0261 (6)	−0.0025 (5)	0.0036 (5)	0.0006 (5)
C27	0.0274 (6)	0.0335 (7)	0.0323 (6)	−0.0013 (5)	0.0054 (5)	0.0005 (5)
C28	0.0258 (6)	0.0304 (6)	0.0327 (6)	−0.0015 (5)	0.0081 (5)	−0.0006 (5)
C29	0.0344 (6)	0.0277 (6)	0.0340 (6)	−0.0014 (5)	0.0082 (5)	−0.0058 (5)
C30	0.0374 (6)	0.0317 (6)	0.0273 (6)	−0.0005 (5)	0.0057 (5)	−0.0038 (5)
C31	0.0288 (6)	0.0280 (6)	0.0313 (6)	−0.0016 (5)	0.0101 (5)	−0.0016 (5)
C32	0.0299 (6)	0.0288 (6)	0.0324 (6)	−0.0027 (5)	0.0085 (5)	−0.0057 (5)
C33	0.0286 (6)	0.0356 (7)	0.0286 (6)	−0.0031 (5)	0.0055 (5)	−0.0035 (5)
C34	0.0446 (8)	0.0504 (9)	0.0359 (7)	0.0018 (6)	−0.0017 (6)	0.0130 (6)
C35	0.0607 (9)	0.0353 (7)	0.0365 (7)	0.0045 (7)	0.0008 (6)	0.0018 (6)
C36	0.0577 (9)	0.0266 (7)	0.0446 (7)	0.0030 (6)	0.0048 (6)	−0.0026 (6)

### Geometric parameters (Å, °)

**Table:**

O1—C1	1.3720 (14)	C14—H14C	0.9800
O1—C14	1.4237 (15)	C15—H15A	0.9800
O2—C34	1.4200 (15)	C15—H15B	0.9800
O2—C21	1.3727 (14)	C15—H15C	0.9800
N1—C7	1.2728 (15)	C16—H16A	0.9800
N1—C4	1.4128 (15)	C16—H16B	0.9800
N2—C16	1.4502 (19)	C16—H16C	0.9800
N2—C11	1.3826 (16)	C21—C22	1.3904 (15)
N2—C15	1.4455 (19)	C21—C26	1.3859 (16)
N3—C24	1.4124 (16)	C22—C23	1.3782 (16)
N3—C27	1.2731 (16)	C23—C24	1.3952 (16)
N4—C31	1.3729 (15)	C24—C25	1.3949 (16)
N4—C35	1.4452 (16)	C25—C26	1.3878 (16)
N4—C36	1.4436 (15)	C27—C28	1.4547 (16)
C1—C6	1.3861 (16)	C28—C29	1.4012 (17)
C1—C2	1.3915 (17)	C28—C33	1.3922 (16)
C2—C3	1.3796 (16)	C29—C30	1.3724 (16)
C3—C4	1.3966 (16)	C30—C31	1.4123 (16)
C4—C5	1.3946 (17)	C31—C32	1.4066 (15)
C5—C6	1.3863 (16)	C32—C33	1.3790 (16)
C7—C8	1.4534 (16)	C22—H22	0.9500
C8—C13	1.3899 (16)	C23—H23	0.9500
C8—C9	1.4028 (17)	C25—H25	0.9500
C9—C10	1.3721 (16)	C26—H26	0.9500
C10—C11	1.4112 (17)	C27—H27	0.9500
C11—C12	1.4048 (18)	C29—H29	0.9500
C12—C13	1.3790 (16)	C30—H30	0.9500
C2—H2	0.9500	C32—H32	0.9500
C3—H3	0.9500	C33—H33	0.9500
C5—H5	0.9500	C34—H34A	0.9800
C6—H6	0.9500	C34—H34B	0.9800
C7—H7	0.9500	C34—H34C	0.9800
C9—H9	0.9500	C35—H35A	0.9800
C10—H10	0.9500	C35—H35B	0.9800
C12—H12	0.9500	C35—H35C	0.9800
C13—H13	0.9500	C36—H36A	0.9800
C14—H14B	0.9800	C36—H36B	0.9800
C14—H14A	0.9800	C36—H36C	0.9800
			
C1—O1—C14	117.38 (9)	H16B—C16—H16C	110.00
C21—O2—C34	117.84 (9)	N2—C16—H16A	109.00
C4—N1—C7	121.70 (10)	N2—C16—H16B	109.00
C11—N2—C16	119.93 (12)	N2—C16—H16C	109.00
C15—N2—C16	115.71 (12)	H16A—C16—H16B	109.00
C11—N2—C15	119.71 (11)	O2—C21—C22	115.04 (10)
C24—N3—C27	121.45 (10)	O2—C21—C26	125.33 (10)
C35—N4—C36	118.17 (10)	C22—C21—C26	119.63 (10)
C31—N4—C35	120.58 (10)	C21—C22—C23	119.86 (10)
C31—N4—C36	120.25 (10)	C22—C23—C24	121.69 (10)
C2—C1—C6	119.61 (11)	N3—C24—C23	115.71 (10)
O1—C1—C6	124.69 (10)	N3—C24—C25	126.68 (10)
O1—C1—C2	115.67 (10)	C23—C24—C25	117.61 (10)
C1—C2—C3	119.80 (11)	C24—C25—C26	121.24 (11)
C2—C3—C4	121.62 (11)	C21—C26—C25	119.96 (10)
N1—C4—C3	115.51 (10)	N3—C27—C28	122.35 (11)
N1—C4—C5	126.83 (10)	C27—C28—C29	122.14 (10)
C3—C4—C5	117.64 (10)	C27—C28—C33	120.46 (11)
C4—C5—C6	121.25 (11)	C29—C28—C33	117.40 (11)
C1—C6—C5	120.03 (11)	C28—C29—C30	121.55 (11)
N1—C7—C8	122.55 (11)	C29—C30—C31	121.12 (11)
C7—C8—C9	122.50 (10)	N4—C31—C30	121.47 (10)
C7—C8—C13	120.38 (11)	N4—C31—C32	121.37 (10)
C9—C8—C13	117.09 (10)	C30—C31—C32	117.16 (10)
C8—C9—C10	121.81 (11)	C31—C32—C33	120.91 (11)
C9—C10—C11	120.98 (11)	C28—C33—C32	121.82 (11)
N2—C11—C12	121.46 (11)	C21—C22—H22	120.00
C10—C11—C12	117.17 (10)	C23—C22—H22	120.00
N2—C11—C10	121.35 (11)	C22—C23—H23	119.00
C11—C12—C13	120.97 (11)	C24—C23—H23	119.00
C8—C13—C12	121.97 (11)	C24—C25—H25	119.00
C1—C2—H2	120.00	C26—C25—H25	119.00
C3—C2—H2	120.00	C21—C26—H26	120.00
C4—C3—H3	119.00	C25—C26—H26	120.00
C2—C3—H3	119.00	N3—C27—H27	119.00
C4—C5—H5	119.00	C28—C27—H27	119.00
C6—C5—H5	119.00	C28—C29—H29	119.00
C5—C6—H6	120.00	C30—C29—H29	119.00
C1—C6—H6	120.00	C29—C30—H30	119.00
C8—C7—H7	119.00	C31—C30—H30	119.00
N1—C7—H7	119.00	C31—C32—H32	120.00
C8—C9—H9	119.00	C33—C32—H32	120.00
C10—C9—H9	119.00	C28—C33—H33	119.00
C11—C10—H10	119.00	C32—C33—H33	119.00
C9—C10—H10	120.00	O2—C34—H34A	109.00
C13—C12—H12	119.00	O2—C34—H34B	109.00
C11—C12—H12	120.00	O2—C34—H34C	110.00
C8—C13—H13	119.00	H34A—C34—H34B	109.00
C12—C13—H13	119.00	H34A—C34—H34C	109.00
O1—C14—H14B	109.00	H34B—C34—H34C	109.00
O1—C14—H14C	109.00	N4—C35—H35A	109.00
O1—C14—H14A	109.00	N4—C35—H35B	109.00
H14A—C14—H14B	110.00	N4—C35—H35C	110.00
H14A—C14—H14C	109.00	H35A—C35—H35B	109.00
H14B—C14—H14C	109.00	H35A—C35—H35C	109.00
N2—C15—H15C	109.00	H35B—C35—H35C	110.00
H15A—C15—H15B	109.00	N4—C36—H36A	109.00
H15A—C15—H15C	109.00	N4—C36—H36B	109.00
N2—C15—H15A	109.00	N4—C36—H36C	110.00
N2—C15—H15B	109.00	H36A—C36—H36B	110.00
H15B—C15—H15C	109.00	H36A—C36—H36C	109.00
H16A—C16—H16C	110.00	H36B—C36—H36C	109.00
			
C14—O1—C1—C2	176.72 (10)	C13—C8—C9—C10	−0.17 (17)
C14—O1—C1—C6	−5.23 (16)	C9—C8—C13—C12	0.37 (17)
C34—O2—C21—C26	−0.55 (17)	C7—C8—C13—C12	−177.57 (11)
C34—O2—C21—C22	179.41 (11)	C8—C9—C10—C11	−0.38 (18)
C4—N1—C7—C8	−177.55 (10)	C9—C10—C11—C12	0.72 (17)
C7—N1—C4—C5	7.05 (18)	C9—C10—C11—N2	179.19 (11)
C7—N1—C4—C3	−174.49 (11)	N2—C11—C12—C13	−179.00 (11)
C15—N2—C11—C10	175.35 (11)	C10—C11—C12—C13	−0.52 (17)
C16—N2—C11—C12	−161.06 (12)	C11—C12—C13—C8	−0.02 (18)
C15—N2—C11—C12	−6.25 (17)	O2—C21—C22—C23	179.55 (10)
C16—N2—C11—C10	20.54 (17)	C26—C21—C22—C23	−0.48 (17)
C27—N3—C24—C25	−14.68 (18)	O2—C21—C26—C25	−179.09 (11)
C27—N3—C24—C23	166.32 (11)	C22—C21—C26—C25	0.95 (17)
C24—N3—C27—C28	−179.28 (10)	C21—C22—C23—C24	−0.35 (18)
C35—N4—C31—C30	10.32 (18)	C22—C23—C24—N3	179.77 (11)
C35—N4—C31—C32	−170.47 (12)	C22—C23—C24—C25	0.68 (17)
C36—N4—C31—C32	−2.15 (18)	N3—C24—C25—C26	−179.18 (11)
C36—N4—C31—C30	178.65 (12)	C23—C24—C25—C26	−0.20 (17)
O1—C1—C6—C5	−176.45 (11)	C24—C25—C26—C21	−0.61 (18)
C2—C1—C6—C5	1.53 (17)	N3—C27—C28—C29	1.76 (18)
C6—C1—C2—C3	−1.72 (17)	N3—C27—C28—C33	−179.18 (11)
O1—C1—C2—C3	176.44 (10)	C27—C28—C29—C30	−179.90 (11)
C1—C2—C3—C4	0.32 (17)	C33—C28—C29—C30	1.01 (18)
C2—C3—C4—C5	1.23 (16)	C27—C28—C33—C32	179.75 (11)
C2—C3—C4—N1	−177.38 (10)	C29—C28—C33—C32	−1.15 (18)
C3—C4—C5—C6	−1.41 (17)	C28—C29—C30—C31	0.70 (19)
N1—C4—C5—C6	177.02 (11)	C29—C30—C31—N4	177.00 (12)
C4—C5—C6—C1	0.06 (18)	C29—C30—C31—C32	−2.24 (18)
N1—C7—C8—C9	−1.15 (18)	N4—C31—C32—C33	−177.13 (11)
N1—C7—C8—C13	176.68 (11)	C30—C31—C32—C33	2.11 (17)
C7—C8—C9—C10	177.73 (11)	C31—C32—C33—C28	−0.44 (18)

### Table 2 Intermolecular C—H···π interactions (Å, °)

**Table:**

D - H···Cg	D—H	H···Cg	D—H···Cg
C3—H3···Cg3	0.95	2.63	152
C6—H6···Cg3^i^	0.95	2.81	142
C10—H10···Cg4	0.95	2.88	145
C13—H13···Cg4^i^	0.95	2.73	137
C30—H30···Cg2^ii^	0.95	2.94	136
C34—H34A···Cg1^iii^	0.95	2.96	114
C36—H36A···Cg1^iv^	0.95	2.91	156

Centroids: Cg1 = ring C1–C6; Cg2 = ring C8–C13; Cg3 = ring C21–C26; Cg4 = ring C28–C33. Symmetry codes: (i) -1+x, y, z; (ii) 1-x,-y,2-z; (iii) 1/2+x,1/2-y,-1/2+z; (iv) 1-x,-y,1-z.
